# The perceptions of professional soccer players on the risk of injury from competition and training on natural grass and 3rd generation artificial turf

**DOI:** 10.1186/2052-1847-6-11

**Published:** 2014-03-01

**Authors:** Constantine CN Poulos, John Gallucci, William H Gage, Joseph Baker, Sebastian Buitrago, Alison K Macpherson

**Affiliations:** 1 School of Kinesiology and Health Science, York University, 4700 Keele St, M3J 1P3 Toronto, Canada; 2 JAG Physical Therapy, New Jersey, USA; 3 Major League Soccer, New York City, USA

**Keywords:** Soccer, Perceptions, Artificial turf, Injury, Grass

## Abstract

**Background:**

The purpose of this study was to describe professional soccer players’ perceptions towards injuries, physical recovery and the effect of surface related factors on injury resulting from soccer participation on 3rd generation artificial turf (FT) compared to natural grass (NG).

**Methods:**

Information was collected through a questionnaire that was completed by 99 professional soccer players from 6 teams competing in Major League Soccer (MLS) during the 2011 season.

**Results:**

The majority (93% and 95%) of the players reported that playing surface type and quality influenced the risk of sustaining an injury. Players believed that playing and training on FT increased the risk of sustaining a non-contact injury as opposed to a contact injury. The players identified three surface related risk factors on FT, which they related to injuries and greater recovery times: 1) Greater surface stiffness 2) Greater surface friction 3) Larger metabolic cost to playing on artificial grounds. Overall, 94% of the players chose FT as the surface most likely to increase the risk of sustaining an injury.

**Conclusions:**

Players believe that the risk of injury differs according to surface type, and that FT is associated with an increased risk of non-contact injury. Future studies should be designed prospectively to systematically track the perceptions of groups of professional players training and competing on FT and NG.

## Background

Participation in soccer poses an inherent risk of injury, that can arise from the interplay of many different factors. The incidence of injury due to soccer participation has been shown to range from 12 to 35.5 injuries/1000 hours of games and 1.5 to 7.6 injuries/1000 of practice for different leagues across the world, and varying levels of competition – mostly adult male professional players (e.g., lower tier professional division, top-tier professional division, international level competition) [1]. It is imperative to understand the risk factors leading to injury in soccer in order to initiate measures to reduce their occurrence and associated burden. Currently, two types of surfaces are sanctioned by FIFA and UEFA for soccer competition at the elite professional level: natural grass (NG) surfaces, and artificial turf surfaces (including both 3rd generation and 4th generation) otherwise referred to as “Football turf” (FT) [2]. The main reasons for the introduction of artificial turf were to lessen the impact of environmental conditions on surfaces, to decrease the high operating costs associated with NG, and to increase field usability [3-5].

Studies analyzing the injury risk associated with elite soccer competition and training on FT and NG have found comparable rates of injury on both surfaces [6-10]. In a recent study of elite male professional players the incidence of acute injuries were reported to be 3.5 v 3.5/1000 training hours, and 22.4 v 21.7/1000 match hours for FT and NG respectively [7]. Similar incidences were further reported in an earlier study of elite male professional players (2.42 v 2.49/1000 training hours and 19.60 v 21.48/1000 match hours for FT and NG respectively) [6]. Notably missing in this body of literature are studies exploring professional soccer players’ subjective experiences about competition and training on FT and NG. No study has focused on understanding professional soccer players’ opinions about the influence of surface type on injury.

Movement patterns, ball skills, and the impressions of Swedish elite male and female soccer players were analyzed during competitive games on 2nd and 3rd generation artificial turf and NG by Andersson et al. [11]. No differences were observed in the movement or technical patterns of the players between the two surfaces, yet roughly two thirds of the male sample reported that games were more physically demanding on artificial turf compared to natural grass [11]. However, Nedelec and colleagues found that a sample of 13 professional players had no negative impressions of artificial turf during recovery (data were collected 10 minutes, 24 hours, and 48 hours after the test) following a 90-minute soccer-specific aerobic field test performed on NG and artificial turf [12]. Players did although report moderately higher soreness in the quadriceps immediately after the test, in the gluteals 24 hours after the test, and in the hamstrings 48 hours after the test in the artificial turf condition [12].

The aim of this study was to assess professional soccer players’ perceptions regarding injuries, physical recovery, and the influence surface related factors such as field mechanical properties and maintenance can have on injury, and other complaints related to soccer participation on FT compared to NG. Doing so is important for assessing the completeness of the current surface-injury paradigm, and to put the findings of the current epidemiological literature into the context of professional players day-to-day experiences with these surfaces, thereby narrowing the gap between science and practice.

## Methods

Major League Soccer is the highest level of professional soccer in North America. It is comprised of 16 teams from the United States and 3 teams from Canada, which compete from March to December. Along with regular season competition, teams compete concurrently in friendly matches, domestic tournaments, and in one major international tournament (CONCACAF Champions League). Of the 18 teams competing in the MLS during the 2011 season, 6 teams were invited to participate in the study based upon the availability and willingness of these teams to participate in the study directives. Access to the teams was facilitated by the Medical Coordinator for the MLS, who provided contact information, and support throughout the study period. Fulltime professional players who were signed to a first team contract were asked to complete a paper questionnaire. All participating players had to be able to read and speak English, and voluntarily consent to participation in the study. Players were instructed to recall their experiences on FT rather than earlier generation turf surfaces, and a description of FT was provided in the survey. In accordance with the UEFA model for epidemiological studies a member of the medical team was selected as the primary contact person [13]. The Head and/or Assistant Athletic Therapist’s/Trainer’s were responsible for administering the surveys. The study was reviewed and approved under the research ethics protocols by the Human Participants Review Subcommittee at York University, Toronto, Canada.

We could not find a validated questionnaire and had therefore developed our own. A graphic artist was hired to design the visual components of the survey, such as the color scheme, text selection and graphical layout. An extensive literature review was conducted to identify: 1) Salient issues in the current research on NG and FT (e.g., more ankle injuries occur on artificial turf, and player impressions that it is harder, and more fatiguing to play on artificial turf as opposed to grass). 2) Outcome variables that have been used in soccer injury research (e.g., contact vs. non-contact injuries, acute vs. chronic injuries, exposure time, age, and anatomical region of injury). Further questions were drawn from the National Football League Players Association (NFLPA) *Playing Surfaces Opinion Survey*, administered to all active NFL players in 2004 and 2008 [14,15]. The final questionnaire was a consolidation of the information from these sources, and included 18 closed-ended attitudinal questions, 2 of which had an open-ended component asking players “why” they chose their respective response, and 1 open-ended attitude question. All technical terms used in this survey were defined following the recommendations of the *Consensus Statement on Injury Definitions and Data Collection Procedures in Studies of Soccer Injuries* published under the auspices of the FIFA Medical Assessment and Research Center [16]. The questionnaire was pilot-tested with the York University Varsity Men’s Soccer Team and minor changes were made before it was administered to the study sample. A copy of the questionnaire administered to the players can be found in Additional file 1 A electronically online.

Some questions in each survey were missing answers. In these cases the completed data were used in the analysis, and the missing data were excluded. Descriptive statistics including means, standard deviations and frequencies, were calculated using SPSS version 20.0 (IBM Corp., Armonk, NY). Open-ended responses were analyzed and interpreted by the primary research team and a Certified Athletic Therapist who works in a soccer setting. Table 1 includes the key phrases in the players’ comments that were used to stratify responses into the surface mechanical groupings.

**Table 1:** Key phrases for mechanical group comments

**Group**	**Key phrases**
Stiffness	The following are “key phrases” in the responses that corresponded with this surface property:
	*“Too hard”, “hard impact”, “unforgiving surface”, “pounds on joints”,* ***“no give in ground”****, “tougher surface”, “firm surface”, “poor shock absorption”*
Friction	The following are “key phrases” in the responses that corresponded with this surface property:
	*“Studs/foot get stuck”,* ***“no give”****, “feet stick”, “cleats don’t slide”, cleats get caught”*
Metabolic cost	The following are “key phrases” in the responses that corresponded with this surface property:
	*“Requires more physical effort”, “running in sand”, “work harder”, “heavy surface”, “body gets tired faster”, “fatigues body“*

## Results

Out of a total of 18 teams in the league at the time of the study 6 teams completed the surveys (33%). Out of a total of 180 potential respondents across the six teams, 99 players (55%) completed the surveys. For the 13 questions analyzed 4% of the data were missing (49/1,396 potential responses). Table 2 provides data on the 99 players and the 6 teams.

**Table 2:** Player and team demographics

**Team**	**Players**	**Age, y**	**Years pro**	**Training surface**	**Competition surface**	**% of career on FT**	**% of career on NT**
1	21	24.4 ± 4.2	4.7 ± 3.7	FT	NG	38	62
2	17	23.1 ± 2.2	3.2 ± 2.7	NG	NG	34	66
3	10	26.5 ± 2.9	6.5 ± 2.4	NG	NG	26	74
4	15	23.6 ± 3.3	3.8 ± 3.2	NG	NG	23	77
5	18	24.0 ± 5.4	4.4 ± 4.6	NG	NG	29	71
6	18	25.9 ± 5.6	5.2 ± 4.7	NG	NG	33	67
Total	99	24.5 ± 4.2	4.5 ± 3.8			35	65

FT, Football Turf. NG, Natural Grass.

### Physical experiences on football turf and natural grass

Most of the players responded that playing and practicing on FT resulted in greater muscle and joint soreness (97%, 96/99 and 96%, 95/98; 1 missing response respectively). Further, the majority of players (90%, 89/99) also felt that it took more time to recover after a game on FT when compared to NG. These findings were also reflected in the following terms, that players used to describe FT; *“Greater Stress of Joints/Body”, “Greater Muscle Tightness/Soreness”, “Breaks down body”, “Shock to Muscles/Joints”*. When asked which surface most impacted overuse injuries over the entire course of a season only 2 players (2%, 2/98; 1 missing response) felt that NG would lead to more chronic injuries, 9 players (9%, 9/98; 1 missing response) were unsure or had no opinion, and the remaining 87 players (89%, 87/98; 1 missing response) chose FT. There was a difference between how players perceived contact and non-contact injuries. Seventy percent of the players (68/98; 1 missing response) agreed that the risk of sustaining a contact injury was the same regardless of the surface the training session or match was being conducted on. Conversely, eighty percent (78/98; 1 missing response) of the players felt the risk of sustaining a non-contact injury was elevated when training and competing on FT. Overall, 94% of the players (92/98; 1 missing response) chose FT as the surface most likely to increase the risk of sustaining an injury.

### Analysis of player comments

The two open-ended questions asked the players’ “why” they chose their respective response to the following closed-ended items: a) “On which surface does it take you more time to recover after a game?” b) “Overall on which surface do you feel the risk of sustaining an injury is higher?” For question (a) there were 17 missing responses (82/99), and for question (b) there were 26 missing responses (73/99). Three surface related risk factors (i.e., surface stiffness, surface friction, and metabolic cost) were identified by analyzing the key phrases in the players’ responses (Table 3).

**Table 3:** Frequency and distribution of mechanical comments for open ended questions

**Nature of player comments**	**On which surface does it take you more time to recover after a game?**	**Overall on which surface do you feel the risk of sustaining and injury is higher?**
Mechanical comments	47(57%)	43(59%)
Other	35(43%)	30(41%)
Total	82(100%)	73(100%)
Mechanical comments		
1. Stiffness	35(75%)	22(51%)
2. Friction	3(6%)	12(28%)
3. Metabolic cost	7(15%)	0(0%)
4. Stiffness and friction	2(4%)	9(21%)
Total	47(100%)	43(100%)

### List of original player comments

The original player comments below provide further insight into the viewpoint of FT for this group of players.

“All my 3 biggest injuries have happened on turf matches, hence why I believe natural grass is still much safer”

“Feet stick when wet, and the ball moves too fast and your joints are put under more stress because there is no give in turf surface”

“Personally coming off back-to-back ACL tears in my left Knee (both happened on turf) I feel mentally scared to play on turf. Even prior to the ACLs I was hesitant to play full out”

“Football Turf doesn’t give like grass. If a foot gets caught in, it is more dangerous because the turf can’t dig up to release the foot”.

“It’s just proven in my experience of playing that more injuries occur on turf. To avoid this the quality of the turf has to be very high”

“I have always played a little cautious on turf for fear of having a cleat stick in the turf”

“The first time I played on the new type of field turf, I broke my 5th metatarsal in a non-contact plant”

“Temperature on hot days zaps and dehydrates the body”, “Hot, wet weather is difficult to play in”

“Turf can feel like running on sand”, “Requires more physical effort”

### Surface type, quality, and weather as a risk factor for injury

Players agreed that both the type and quality of a playing surface could impact the risk of sustaining an injury (92/99, 93% and 93/98; 1 missing response, 95% respectively). Thirty seven percent of the players (37/99) felt surface quality had the greatest influence on the risk of injury on FT, 36% (36/99) felt the risk of injury was greater on NG, and 21% (21/99) thought that surface quality was no more important in affecting the risk of injury on one surface over the other. The remainder of the players were either unsure or did not have an opinion (5%, 5/99). Sixty eight percent (67/99) of players agreed that climatic conditions can affect the risk of injury on both surfaces. From this group of respondents 48% (32/67) felt that weather affected the risk of injury more on FT, 33% (22/27) felt the risk was the same on both surfaces, 15% (10/67) felt the risk was greater on NG, and 4% (3/67) were not sure. According to the players who reported weather as having an affect on FT 72% (23/32) reported that wet weather had the most significant affect, 19% (6/32) reported hot weather, and 9% (3/32) reported cold weather.

### Age, training surface, and surface history

Three descriptive variables were selected to analyze the players responses across the items: Age (Stratified into three groups: 18 – 22, 23 – 27, and 28+), Training Surface (Team 1 who trained on Football Turf was compared to Teams 2 – 6 who trained on grass), and Surface History (Those players who had a surface exposure of 50% or greater on Football Turf over the course of their careers were compared to those who had less than 50% exposure). A Chi Square test was performed and no significant differences were found between the three descriptive variables and the question responses.

## Discussion

Our findings indicate that a selected group of professional players, representing a sample of professional soccer players in North America believe that there is an increased risk of injury, specifically non-contact injury, as a result of training and competing on FT compared to NG. Previous studies comparing the incidence of injury on FT and NG, found no differences in the risk of injury from training and competition on both surfaces [6-10,17,18]; however 94% of the players in this study felt that the risk of injury was greater on FT. Similarly, players strongly believed that they experienced greater muscle and joint soreness and longer recovery times after competition and training on FT. Three surface mechanical properties (i.e., surface stiffness, surface friction, and metabolic cost) were identified by the players as important factors in surface related injury. Furthermore players’ reported that the magnitudes of the three surface variables were greater on FT, and that these differences were the primary reason why they perceived injury rates, muscle and joint soreness and recovery times to be higher on FT. Along with these three factors players further believed that surface quality and climatic conditions could influence the risk of injury on FT and NG.

A pre-established bias towards synthetic surfaces could possibly explain the divergence of players’ perceptions. Player comments (see the ‘List of original player comments’ section) suggest that past personal injury experiences on FT can mar players’ attitudes toward the surface, and even affect the way they play on FT in the future. Players could have solidified their perceptions of FT based on previous negative experiences on earlier generation turfs that were shown to increase the risk of injury [19]. However, it is unlikely that such experiences and opinions can fully explain why the majority of the players reported greater risk of injury on FT. Moreover players reported that surface type did not influence contact injuries. In an injury audit of 12 European Championships from 2006 to 2008 it was found that traumatic injuries due to player contact represented 54% of all injuries overall, and were more frequent among match injuries (63%) [20]. In order to understand how surface type might affect these injuries future comparative studies should report the occurrence, and mechanisms of both non-contact and contact injuries.

In the NFLPA Surface Opinion Survey conducted in 2004 and 2008 it was found that 96% and 91% of all NFL players reported feeling more soreness and fatigue on artificial in-filled surfaces as opposed to grass [14,15]. These results are similar to those found for the group of players in this study and it would be interesting to see if these findings extend to the entire MLS; and further, to other professional soccer leagues in North America and internationally. The findings of this study suggest that the full effects of training and competing on FT have not been captured in the current literature. This might be related to the definition of injury (i.e., using the time-loss definition, which according to the consensus statement on injury only records an event as an injury if a player cannot take full part in future training or match play). This method is not sensitive enough to capture self-reported problems, such as players experiencing soreness, as they would still participate in sessions or games. This notion is supported by Walden et al., who in a study of injuries in Swedish Elite Football suggested that subjective somatic complaints without objective signs of injury might not be captured by certain injury definitions [21]. It is possible that these effects (i.e., greater muscle and joint soreness after games and training) could also explain why players perceive the risk of injury to be higher on FT. Future epidemiological studies using a time-loss definition should prospectively track players perceptions, specifically levels of muscle and joint soreness and recovery times, concurrently with injury incidence for players training and competing on both surfaces. Doing so will provide another level of comparison between NG and FT, and could potentially uncover novel information on the dynamics between soreness, recovery, and injury for professional players over a period of competition and training regardless of, or in light of surface type.

Surprisingly players identified without any prompt, the surface mechanical properties reported in the literature as risk factors modifying the risk of injury on both surfaces [10]. Greater surface stiffness seems to be the primary reason why players in this study reported the risk of injury to be higher on FT, as it was reported with the greatest frequency. It is speculated that the stiffness properties of a surface influence the frequency of injury and that harder surfaces can increase the impact forces on the body, which in turn might have an influence on some chronic overuse injuries [5]. Similar results have been reported in a study by Martinez et al., in which players reported that artificial turf had worse shock absorbency properties than NG [22]. Similarly, 57% of all NFL players in 2008 believed that new artificial infilled surfaces should be made softer, and 92% reported that they could distinguish the difference between a softer or firmer artificial surface [15]. The player comments also seem to support the postulation that high frictional forces between the foot and playing surface results in foot fixation and possibly injury [23]. Evidence of higher physiological activation on FT found in the literature can also put the players comments into context [24]. Similar findings were also reported by Andersson et al. who found that male elite players reported games on artificial turf as more physically demanding compared to natural grass [11]. Fatigue has been associated with an increase of injury in soccer players [10], and therefore the reported perception of higher physiological activation on FT in this study, and others, could be a contributing mechanism to injury risk. Based on these findings more research needs to be undertaken to understand the player-surface relationship and how it possibly influences the risk of injury. In particular future studies should focus attention on how surface stiffness and injury risk are related on FT and NG, as it seems that independent groups of professional players (NFL American football players, soccer players) have the opinion that FT is “too hard”.

Although it is widely accepted that without proper maintenance the performance and physical characteristics of FT decline, it is unknown if a decline in surface quality can affect the risk of injury. The fact that players in this study reported surface quality as being important in affecting the risk of injury for both NG and FT suggests that there could be a link between these two variables. It follows that proper maintenance of FT and NG is important to players, and that perhaps the relationship between the risk of non-contact injury and the quality of FT should be explored in future studies.

Climate and weather conditions can have a significant influence on the playing conditions of NG, and this has been shown to impact sport related injury [25]. Considering this, it is perplexing that players in this study believed weather to have a greater influence in affecting injury on FT. Although FT retains a significant amount of heat in hot weather, wet weather was reported in the greatest frequency for affecting injury on FT. A possible explanation for this finding is that wet weather accelerates the movement of the ball, thereby the speed of play, more so on FT than NG forcing players to work harder and imposing increased strain on the body to compete in such an environment. This postulation is supported by the opinions of the expert group of players and coaches interviewed in a study by Martinez et al., who found ball roll to be more rapid on artificial surfaces [22]. Further evidence into how speed of play could affect the risk of injury on FT can be found in the original player comments section.

It has been suggested that familiarization with FT is important to consider when measuring players impressions of artificial and natural grounds [12]. In a study by Nedelec et al. the absence of negative perceptions of FT by a group of young male professional soccer players was explained to be in part due, to the familiarization of FT for this group of players [12]. In contrast, in the present study it was found that players who had a history of playing on FT and players who currently trained on FT expressed preferences for NG, and had negative impressions of FT. The divergence of our findings and those of Nedelec et al. could be due to the age of the players employed in each study (average age 17.7 v 24.5 years for the study by Nedelec et al. and the current study respectively). Although we did not observe any significant differences in players’ opinions across the 3 age cohorts, it may be possible that a difference exists in how younger and older professional players perceive FT and NG. Future studies should aim to elucidate how familiarization to FT and age may impact players perceptions of FT.

Practitioners working with male professional soccer players could use the findings of this study to help them manage their players after exposure to FT and to make appropriate decisions for future sessions knowing that players may possibly have longer recovery times and experience greater soreness. This could be especially true when dealing with players who have a history of muscular-tendon injury, past injuries to weight bearing joints, degenerative changes in weight bearing joints, or a history of recurring injury, although this conjecture is unsubstantiated and would need to be explored. Lastly another practical application could be in making appropriate decisions surrounding surface type exposure for various activities when returning a player back from injury in different steps of the rehabilitation process.

There are a number of limitations of this study, which should be noted. The sample size was small and therefore the results may not be representative of the opinions of all MLS players and the much broader, and more diverse, international professional male soccer population as a whole. Furthermore participants were not selected randomly, which could also affect the generalizability of the findings. Recall of information could have been inaccurate due to the study design, which was cross-sectional and retrospective. Players’ responses could have been biased due to previous negative experiences on artificial surfaces, in addition to the cultural stigma surrounding artificial surfaces in soccer. Lastly, this study looked at professional male soccer players and therefore the results may not be characteristic of the opinions of sub-elite or amateur male players and elite, sub-elite or amateur female players. In a study by Zanetti it was found that Italian male amateur soccer players preferred playing on 3rd generation artificial turf rather than natural grounds [26], and Andersson et al. found elite female players reported a neutral position towards artificial grounds. Further research needs to be conducted on these populations [11].

## Conclusion

This study explored the perceptions of injury, physical recovery, as well as the effect of surface related variables on injury as a result of soccer participation on NG and FT for 99 professional soccer players competing in Major League Soccer during the 2011 season. Overall it was found that players perceived FT as the surface most likely to lead to injury, more specifically non-contact injury, was associated with longer recovery times after games and training, as well as greater muscle and joint soreness.

## Competing interests

The authors declare that they have no competing interests.

## Authors’ contributions

CCNP designed the study, developed the questionnaire, lead the data collection process, analyzed the data, and drafted the manuscript. JG Jr. provided access to the participating teams, aided in the data collection process, and aided in the editing of the manuscript. WHG provided insight into the questionnaire and study design, and aided in the drafting of the manuscript. JB aided in the drafting of the manuscript. SB aided in the questionnaire design, data analysis, and aided in the editing of the manuscript. AKM oversaw the study, providing insight into the study and questionnaire design, aiding in the data analysis, and drafting of the manuscript. All authors read and approved the final manuscript.

## Pre-publication history

The pre-publication history for this paper can be accessed here:

http://www.biomedcentral.com/2052-1847/6/11/prepub

## Supplementary Material

Additional file 1 Players’ Surface Opinion Survey on Natural Grass and Artificial Turf: AFocus on Injury. Click here for file
