# Supplementary material for: The perceptions of professional soccer players on the risk of injury from competition and training on natural grass and 3rd generation artificial turf
Source: BMC Sports Sci Med Rehabil. 2014 Mar 1;6:11. doi: 10.1186/2052-1847-6-11 (PMC4108054; doi:10.1186/2052-1847-6-11)
Supplement: Additional file 1 — Players’ Surface Opinion Survey on Natural Grass and Artificial Turf: AFocus on Injury. [file 2052-1847-6-11-S1.pdf]

PLAYERS' SURFACE OPINION SURVEY ON NATURAL GRASS AND ARTIFICIAL TURF: A FOCUS ON INJURY

Dear Athlete,

What you have just been handed is a questionnaire about your opinions of Natural Grass and Artificial Turf and how they affect your risk of injury while playing soccer. My name is Constantine Poulos, and I am a Masters student at York University, in Toronto Canada. This is my project that I have decided to do for my thesis. Along with myself the other two researchers are Sebastian Buitrago and John Gallucci. We are interested in professional soccer players' opinions of how surface type (Natural Grass and Artificial Turf) could affect the risk of injury, and so we are asking you about what you think. This questionnaire should take 20 to 30 minutes to complete and there are three parts that you have to go through if you decided to participate:

1. **Informed Consent Form**
2. **Player Profile**
3. **Players Surface Opinion Survey**

I would like to thank you for taking the time to read this. Your opinion is extremely valuable, and your help would be very much appreciated. If you have any questions or concerns please do not hesitate to contact me at any time, and I will makes sure to get back to you. Good luck with the rest of the season, and I wish you all success!

Regards,

Constantine Poulos, ATTH, CSCS MSc. Candidate, CAT(C) Candidate School of Kinesiology and Health Science
York University, Toronto, Canada Tel: (416) 736-2100 ext. 20229 Email: kostap@yorku.ca Twitter: @Kosta_Poulos

YORK U
UNIVERSITÉ
UNIVERSITY
redefine THE POSSIBLE.

PART 1
INFORMED CONSENT FORM

The purpose of this study is to collect and describe professional soccer players' opinions of how surface type affects their perceived risk of sustaining an injury. The two surfaces that are being investigated are natural grass and 3rd generation Football turf, which is the newest type of artificial turf, used for professional soccer competition.

To collect this information you will be invited to complete a questionnaire that will be administered by your teams' medical staff. Each questionnaire will consist of instructions and a set of questions. The survey will consist of a combination of multiple choice and short answer questions, and should take approximately 20 to 30 minutes to complete. The questions can be found in the survey booklet you have received with this form. Participation in this survey is voluntary and you can withdraw your co-operation at any time. If you choose to do so, any data that you may have provided will be destroyed and not be used in the project.

There is no know harm in your participation in this survey. You might potentially benefit by being able to express your opinion on a matter that is important to you as a professional soccer player. You will not receive any incentives for completing this survey.

Confidentiality will be respected and no information that discloses the identity of you, the participant will be released or published. At no point during the analysis and presentation of the findings of this survey will names of players, and teams be mentioned. You will not be required to write your name on the survey, and any screening questions will not reveal your identity. Consent forms will be separated from the completed survey so that the primary researcher, and any second party will not be able to discover your identity.

All consent forms and completed surveys will be mailed back to the primary researcher and stored in a secured locked area to which only the researchers have access. These documents will be stored for roughly 1.5 years during the duration of the project, and then shredded.

This project has been reviewed and approved under research ethics protocols by the Human Participants Review Subcommittee (HPRC) of York University. If you have any questions or concerns you can contact the Graduate Program Office of York University by phone: 416-736-5728 or by email: kahs@yorku.ca. If you have any concerns regarding the ethics of this project please contact Alison Colins-Mrakas, the Senior Manager and Policy Advisor at the Office of Research and Ethics by phone at 416-736-5914.

Thank you very much for taking the time to read this document. If you have decided to complete the survey please provide your signature and date in the space provided below, and continue to the next page. If you have decided to not participate your consideration is much appreciated, and you can stop the survey now. Please feel free to contact me, Constantine Poulos, the primary researcher at anytime. Best of luck in the remainder of the season, and I hope this letter finds you well.

Constantine Poulos

Date

Player signature

Date

THANK YOU

PART 2
PLAYER PROFILE

The purpose of filling out this brief player profile is to be able to match your responses witch certain things like how many years you have been in the league, or how old you are. For example, we would use this information to look at question like "do older players feel artificial turf causes more injuries than younger players?" These questions are not meant to identify you in any way.

How old are you?

....

What position do you play?

....

How many years have you been playing professional soccer?

....

What type of surface do you currently train on?

....

What type of surface is your home field?

....

Over your entire playing career (amatuer to professional) how much time have you spent training and competing on both surfaces? Please place an X along the line in a position that best shows your soccer history on these two surfaces.

100% on Football Turf	50% on Football Turf 50% on Grass	100% on Natural Grass

PART 3
PLAYERS' OPINION SURVEY

This is the last part of the survey, and the most important. What comes next is a set of brief instructions that you must read before completing the survey. There is also a definitions list, of terms used in the survey that you can refer back to if you are unsure of a term.

Instructions

- Please use pen to mark the survey.
- Please circle the letter of the response that you choose.
- For the questions that you need to write your opinion only use as much space as you need. You do not need to fill in all the blank space.
- Football Turf refers to the newest type of artificial turf, with the long artificial grass with the rubber pellets. Always make sure to think about that turf when answering the questions.
- If you have any questions or are confused you can ask your Athletic Therapist/Trainer, but please don't ask your teammates.

Definitions

Injury - Any physical complaint sustained by a player that results from a soccer match or soccer training, regardless of the need for medical attention or time-loss from soccer activities.

Football Turf - The newest type of artificial turf used currently in professional soccer.

Recurrent Injury - An injury of the same type and at the same site as an initial injury and which occurs after a players' return to full participation from the initial injury.

Traumatic Injury - An injury resulting from a specific, know event. Ex. Kicked in the shin.

Chronic overuse Injury - An injury caused by repeated microtrauma without a single, know event responsible for the injury. Ex. Low back is consistently painful, but not sure from what.

Contact Injury - Traumatic injury resulting from contact with another player or object.

Non-Contact Injury - An injury that occurs with no physical contact with other players' or object at the time of the incident. Ex. Knee Ligament sprain while cutting during play.

Player Survey

1. The type of surface that you train and play on is an important factor in determining the risk of sustaining an injury.

a) Strongly agree
b) Agree
c) Neither agree or disagree
d) Disagree
e) Strongly disagree
f) Don't know

2. Does weather affect the risk of sustaining an injury on both Football Turf and Natural Grass?

a) Yes
b) No
c) Not sure
d) No opinion

If you answered (a) then continue to question 3. If you answered, (b), (c), or (d) then move on to question 5.

3. On which surface does weather affect the risk of injury the most?

a) Natural Grass
b) Football Turf
c) Risk is the same on both
d) Not Sure
e) No Opinion

If you answered (a) or (b) then move on to question 4. If you answered (c), (d), or (e) then move on to question 5.

4. Based on your response to question 3, which weather condition increases the risk of injury on that surface the most?

a) Wet weather
b) Hot weather
c) Cold weather
d) Windy weather

5. The quality of a playing surface affects the risk of injury while playing and training on that surface.

a) Strongly Agree
b) Agreee
c) Neither agree or disagree
d) Disagree
e) Strongly disagree
f) Don't know

6. On which surface does field quality (Ex. Maintenance, Pitch Condition) have the biggest impact in affecting the risk of injury?

a) Natural Grass
b) Football Turf
c) Risk is the same on both
d) Not sure
e) No opinion

7. Which surface do you feel leads to greater muscle and joint soreness after a game?

a) Natural Grass
b) Football Turf
c) Feel the same on both
d) Not sure
e) No opinion

8. Which surface do you feel leads to greater muscle and joint soreness after a training session?

a) Natural Grass
b) Football Turf
c) Feel the same on both
d) Not sure
e) No opinion

9. On which surface does it take you more time to recover after a game?

a) Natural Grass
b) Football Turf
c) Recovery is the same on both
d) Not sure
e) No opinion

If you answered (a), or (b) then please explain why in the space below. If you answered (c), (d), or (e) then please move on to question 10.

....
....
....
....
....
....

10. Do you believe that surface type can affect the risk of sustaining an overuse injury during the preseason?

a) Yes
b) No
c) Surface type doesn't affect overuse injuries
d) Not sure
e) No opinion

If you answered (a) then please move on to question 11. If you answered (b), (c), (d), or (e) then please move on to question 12.

11. What surface do you think is more likely to cause an overuse injury during the preseason?

a) Natural Grass
b) Football Turf
c) Other ....

12. If you are coming off an injury, the likelihood of a re-injury occurring is greater if upcoming training sessions are scheduled on which of the following surfaces?

a) Natural grass
b) Football Turf
c) Risk is the same on both surfaces
d) Surface type doesn't affect the occurrence of a re-injury
e) No Sure
f) No Opinion

13. The risk of sustaining a contact injury (Ex. Player-to-player contact) is the same regardless of the surface that the training session or match is being played on.

a) Strongly agree
b) Agree
c) Neither agree or disagree
d) Disagree
e) Strongly disagree
f) Don't know

14. Which surface do you feel increases the risk of non-contact injuries during training and match play (Ex. Knee ligament sprain while cutting)?

a) Natural grass
b) Football Turf
c) No difference between surfaces
d) Not sure
e) No opinion

15. The risk of sustaining a non-contact ankle sprain is the same on Natural grass or Football Turf.

a) Strongly Agree
b) Agree
c) Neither agree or disagree
d) Disagree
e) Strongly Disagree
f) Don't Know

16. On which surface do you feel the risk of sustaining a non-contact ankle sprain is greater?

a) Natural Grass
b) Football Turf
c) Risk is the same on both surfaces
d) Not Sure
e) No Opinion

17. Which surface over the course of a season most influences the development of chronic overuse injuries?

a) Natural Grass
b) Football Turf
c) Surface type doesn't chronic overuse injuries
d) Not Sure
e) No Opinion

18. Overall on which surface do you feel the risk of sustaining an injury is higher?

a) Natural Grass
b) Football Turf
c) Risk is the same on both
d) Not Sure
e) No Opinion

If you answered (a) or (b) then please explain why in the space provided. If you answered (c), (d), or (e) then move on to question 19.

....
....
....
....
....
....

19. Please write any other comments related to how you believe surface type (Natural Grass or Football Turf) affects the risk of injury in Soccer.

....
....
....
....
....
....

THANK YOU
